# A rapid, sensitive, reproducible and cost-effective method for mutation profiling of colon cancer and metastatic lymph nodes

**DOI:** 10.1186/1471-2407-10-101

**Published:** 2010-03-16

**Authors:** Debora Fumagalli, Patrick G Gavin, Yusuke Taniyama, Seung-Il Kim, Hyun-Joo Choi, Soonmyung Paik, Katherine L Pogue-Geile

**Affiliations:** 1 Department of Pathology, National Surgical Adjuvant Breast and Bowel Project (NSABP), 1307 Federal St, Pittsburgh, PA 15212, USA

## Abstract

**Background:**

An increasing number of studies show that genetic markers can aid in refining prognostic information and predicting the benefit from systemic therapy. Our goal was to develop a high throughput, cost-effective and simple methodology for the detection of clinically relevant hot spot mutations in colon cancer.

**Methods:**

The Maldi-Tof mass spectrometry platform and OncoCarta panel from Sequenom were used to profile 239 colon cancers and 39 metastatic lymph nodes from NSABP clinical trial C-07 utilizing routinely processed FFPET (formalin-fixed paraffin-embedded tissue).

**Results:**

Among the 238 common hot-spot cancer mutations in 19 genes interrogated by the OncoCarta panel, mutations were detected in 7 different genes at 26 different nucleotide positions in our colon cancer samples. Twenty-four assays that detected mutations in more than 1% of the samples were reconfigured into a new multiplexed panel, termed here as ColoCarta. Mutation profiling was repeated on 32 mutant samples using ColoCarta and the results were identical to results with OncoCarta, demonstrating that this methodology was reproducible. Further evidence demonstrating the validity of the data was the fact that the mutation frequencies of the most common colon cancer mutations were similar to the COSMIC (Catalog of Somatic Mutations in Cancer) database. The frequencies were 43.5% for *KRAS*, 20.1% for *PIK3CA*, and 12.1% for *BRAF*. In addition, infrequent mutations in *NRAS*, *AKT1*, *ABL1*, and *MET *were detected. Mutation profiling of metastatic lymph nodes and their corresponding primary tumors showed that they were 89.7% concordant. All mutations found in the lymph nodes were also found in the corresponding primary tumors, but in 4 cases a mutation was present in the primary tumor only.

**Conclusions:**

This study describes a high throughput technology that can be used to interrogate DNAs isolated from routinely processed FFPET and identifies the specific mutations that are common to colon cancer. The development of this technology and the ColoCarta panel may provide a mechanism for rapid screening of mutations in clinically relevant genes like *KRAS*, *PIK3CA*, and *BRAF*.

**Trial Registration:**

**ClinicalTrials.gov: **NSABP C-07: NCT00004931

## Background

Recent evidence suggests that mutation profiling can assist in the prognosis and prediction for colon cancer. *KRAS*, *PIK3CA *and *BRAF *mutations are frequent in tumors of the colon and have been associated with poor prognosis [1-6]. However, these results remain controversial because other studies have shown that mutations in these genes are not prognostic. A large study, a meta analysis, of *KRAS *mutations, found that only *KRAS*G12V was a bad prognostic marker; other *KRAS *mutations were not associated with bad prognosis [2]. Evidence has also demonstrated that *KRAS *mutations are potential markers for prediction because tumors with *KRAS *mutations are significantly associated with resistance to EGFR antibody based therapies [7-11]. Publications have reported the same phenomenon with *BRAF *and *PIK3CA *mutations, although these observations are still not well established [12,13]. The published study suggesting that *BRAF *mutant tumors were resistant to EGFR therapies was a small study [13]. Predictive value of *PIK3CA *mutations remains controversial in that other publications have shown that these mutations have no predictive value [14,15]. These inconsistencies, together with two other factors, have limited the impact of mutation profiling for prognosis and prediction in standard care of colon cancer. A large sample size is required to establish that a gene mutation has a significant impact for patient prediction or prognosis. Another limitation is that until recently conducting such large studies with the standard sequencing technologies was too time consuming and too expensive to be practical for clinical studies.

Moreover, while the high frequency of *KRAS*, *BRAF *and *PIK3CA *mutations in colon cancer is well documented, other potentially important mutations have not been profiled with a large number of clinical samples. Whole genome sequencing of a small number of colon samples demonstrated that somatic cancer mutations consist of a few genes that occur frequently and many more mutations that occur very infrequently in many different genes [16,17]. Mutations in these infrequently mutated genes could have a similar effect or synergize with mutations in *KRAS*, *PIK3CA*, and *BRAF*.

Given these considerations, our goal was to find a cost-effective and high throughput methodology that would detect frequent and infrequent cancer mutations genes in a large number of samples. Furthermore, it was essential that the methodology would work with degraded DNAs isolated from FFPET.

The mass spectrometric SNP genotyping technology based on the Sequenom platform provided an ideal choice for mutation profiling to address these several criteria. It has been shown to work with small amounts of degraded DNAs (5 ng), and the high multiplexing capacity minimizes the use of irreplaceable clinical samples. In addition, a variety of studies have demonstrated that the sensitivity of mass spectrometric methods exceeds that of traditional Sanger sequencing and is highly concordant with Sanger sequencing, Pyrosequencing, and allele-specific PCR [16,18,19]. Furthermore, Sequenom has recently developed the OncoCarta Panel, an oncogene panel that offers a rapid and parallel analysis of 238 simple and complex cancer mutations across 19 genes.

The OncoCarta panel includes assays for most colon cancer mutations in the clinically relevant genes, *BRAF *(99%), *KRAS *(98%), and *PIK3CA *(78%), and in addition contains assays for other cancer mutations in genes that intersect with the same pathways as that of *KRAS*, *BRAF*, and *PIK3CA*, such as *AKT1*, *EGFR*, *HRAS*, *NRAS*, *MET *and others. The frequency of *KRAS*, *PIK3CA*, and *BRAF *mutations in the National Surgical Adjuvant Breast and Bowel Project (NSABP) trial C-07 were similar to the frequencies for colon samples listed in the COSMIC data base. This observation provides evidence that the mutation data obtained with the Sequenom platform is accurate. Our results also demonstrate that a majority of colon cancer samples have aberrant *PIK(3) *- *RAS/RAF *network; similar results have been seen previously [6].

Mutations in *ABL1 *and *MET*, not previously identified in colon cancer, were identified, and 13 other genes were screened and found not to be mutated in hot spot locations. Furthermore, this study identified the most frequent colon cancer mutations from OncoCarta, providing the necessary information to reduce the number of assays from 187 to 24, creating a smaller, more specific and economical panel, requiring less DNA, and thus conserving precious clinical samples.

## Methods

### Clinical samples and histological evaluation

Samples used in this study were from NSABP clinical trial C-07. This trial enrolled patients between 02/2000 and 11/2002 to compare oxaliplatin and bolus 5-FU/LV to bolus 5FU/LV alone for resected stage II and III colon cancer [20]. Tissue samples were obtained at surgery before the patients had received any treatment and were routinely processed with FFPE. C-07 was approved by an institutional review board, and informed consent was obtained from each subject. A pathologist categorized tumors into poor, moderate, well differentiated and signet cells carcinoma, according to the World Health Organization (WHO) criteria. Samples were graded for mucinous character, based on the amount of mucin retained within the tumor (1 = no mucin, 2 = < 50% mucinous volume/total tumor volume, 3 = >50% mucinous volume/total tumor volume). Only grade 3 tumors were considered mucinous tumors carcinoma, which is in accordance with WHO criteria.

### DNA isolation

DNA was isolated from FFPE tumor blocks collected from patients participating in NSABP clinical trial C-07. FFPE tumor blocks were cut, and sections of the slide containing the most tumor cells were defined by a pathologist and isolated by macrodissection. Genomic DNA was extracted from 4 five μm unstained sections. After attempting several extraction procedures from a variety of manufacturers (Machery Negel, Qiagen, Ambion), it was determined that the Ambion RecoverAll™ Total Nucleic Acid isolation kit (Applied Biosystem, Foster City, CA) yielded the best DNA based on the quality, quantity, and the performance on the mass spectrometer (data not shown). The extraction was performed as recommended by the manufacturer, with two exceptions; the protease digestion was extended overnight and the elution volume was increased to 150 ul to maximize the total amount of DNA recovered. Additional protease was added to samples incompletely digested after the overnight treatment. DNA was measured with fluorescence, using the Quant-iT ™ PicoGreen^® ^dsDNA Assay Kit (Invitrogen, Carlsbad, CA) and the InfiniteF200 fluorometer (Tecan, Mannedorf, Switzerland).

As a positive control for known mutations and to test the performance of the platform, annotated cell line DNAs (A2058, HS578T, HL60, MCF7, MDAMB231, NCI-H1299, NCI-H1395, UACC-893) were purchased from American Type Culture Collection (ATCC, Manassas, VA, US). Two cell lines, SKBR3 and MCF-7, were grown in culture and cell pellets were fixed in formalin, andembedded in paraffin and DNAs were isolated as described for the clinical samples.

### Mass Spec Type Plex Technology and the OncoCarta Panel

**Figure 1:**
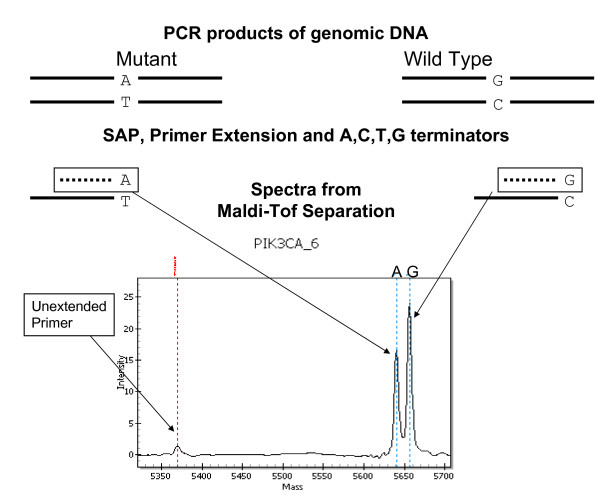
**Methodology for mutation detection**. Genomic DNA from the samples is amplified by PCR, resulting in copies of both mutant and wildtype alleles. Shrimp Alkaline Phosphatase removed excess nucleotides from the sample wells. Primer extension was performed using terminator nucleotides A, C, T, G, each with distinct masses. This linear amplification results in sequences proportional to the alleles that can be distinguished by mass spec (Maldi-Tof Separation).

For mutation detection, the Sequenom platform and the OncoCarta mutation panel were used and the protocol provided by Sequenom (San Diego, CA) was followed with minor modifications. A schematic of the procedure is shown in Fig. 1. A Tecan Evo liquid handler was used to normalize the DNA samples and to set up the PCR reactions. The amount of DNA added to the PCR was reduced to 15 ng or less. DNAs were amplified using the OncoCarta PCR primer pools, unincorporated nucleotides were inactivated by shrimp alkaline phosphatase (SAP), and a single base extension reaction was performed using extension primers that hybridize immediately adjacent to the mutations and a custom mixture of nucleotides. Salts were removed by the addition of a cation exchange resin. Multiplexed reactions were spotted onto the SpectroChipII, and mutations, if present, were resolved by MALDI-TOF on the Compact Mass Spectrometer (Sequenom, San Diego, CA).

The OncoCarta™ Panel v1.0 (Sequenom, San Diego, CA) consists of 24 pools of primer pairs and 24 pools of extension primers, and has the capacity to detect 238 mutations in 19 genes, listed in Table 1. Each pool consists of 5-9 primer pairs in the PCR reaction. Two types of assays have been designed in the OncoCarta panel, referred to as simple and complex. The simple assays are those in which a single assay is able to detect the amino acid changes at that codon. The complex assays are those that require more than one assay to identify codon changes or deletions and insertion, and thus are able to detect multiple different amino acid substitutions or deletions. An example of a complex assay is *KRAS_*1 and *KRAS*_2, which interrogate 2 different nucleotide positions within codon 12 and together identify all codon 12 amino acid changes. Much more complex assays are included in OncoCarta, which interrogate insertions and deletions within the *EGFR *gene.

**Table 1:** Mutations detected with OncoCarta

*ABL1*-G250E	*EGFR*-L747_E749del, A750P	KIT-P585P
*ABL1*-Q252H	*EGFR*-E746_A750del	KIT-D579del
*ABL1*-Y253H	*EGFR*-L747_E749del, A750P	KIT-K642E
*ABL1*-Y253F	*EGFR*-L747_S752del, P753S	KIT-D816V
*ABL1*-E255K	*EGFR*-E746_T751del, V ins	KIT-D816H/D816Y
*ABL1*-E255V	*EGFR*-L747_S752del, Q ins	KIT-V825A
*ABL1*-D276G	*EGFR*-L747_S752del, Q ins	KIT-E839K
*ABL1*-F311L	*EGFR*-E746_T751del, S752D/SNP C2255T	KIT-M552L
*ABL1*-T315I	*EGFR*-D770_N771>AGG/V769_D770insASV/V769_D770insASV	KIT-Y568D
*ABL1*-F317L	*EGFR*-D770_N771insG	KIT-F584S
*ABL1*-M351T	*EGFR*-L747_T750del, P ins	KIT-P551_V555del
*ABL1*-E355G	*EGFR*-E746_A750del	KIT-P551_V555del
*ABL1*-F359V	*EGFR*-E746_T751del, I ins	KIT-Y553_Q556del
*ABL1*-H396R	*EGFR*-L747_T751del	KIT-Y553_Q556del
*AKT1*-rs11555435	*EGFR*-L747_T751del	*KRAS*-G12V/A/D/C/S/R/F
*AKT1*-rs11555431	*EGFR*-E746_A750del, V ins	*KRAS*-G13C/S/V/D
*AKT1*-rs11555432	*EGFR*-E746_A750del, V ins	*KRAS*-A59T
*AKT1*-rs12881616	*EGFR*-S752_I759del	*KRAS*-Q61E/K/L/R/P/H
*AKT1*-rs11555433	ERBB2-L755P	*MET*-R970C
*AKT1*-rs11555436	ERBB2-G776S/G776LC	*MET*-T992I
*AKT1*-rs34409589	ERBB2-G776VC	*MET*-Y1230C
AKT2-S302G	ERBB2-G776VC/G776VC	*MET*-Y1235D
AKT2-R371H	ERBB2-M774_A775insYVMA	*MET*-M1250T
*BRAF*-G464R	ERBB2-A775_G776insYVMA	*NRAS*-G12V/G12A/G12D
*BRAF*-G464V/G464E	ERBB2-P780_Y781insGSP	*NRAS*-G12C/G12R/G12S
*BRAF*-G466V/G466G/G466E	ERBB2-P780_Y781insGSP	*NRAS*-G13V/G13A/G13D
*BRAF*-G466R	ERBB2-S779_P780insVGS	*NRAS*-G13C/G13R/G13S
*BRAF*-F468C	FGFR1-S125L	*NRAS*-A18T
*BRAF*-G469S/E/A/V/R	FGFR1-P252T	*NRAS*-Q61L/Q61R/Q61P
*BRAF*-D594V| G	FGFR3-R248C	*NRAS*-Q61H
*BRAF*-F595L	FGFR3-S249C	*NRAS*-Q61E/Q61K
*BRAF*-G596R	FGFR3-G370C	PDGFRA-V561D
*BRAF*-L597S/R/Q/V	FGFR3-Y373C	PDGFRA-T674I
*BRAF*-T599I	FGFR3-A391E	PDGFRA-F808L
*BRAF*-V600E/K/R/L	FGFR3-K650Q/E	PDGFRA-D846Y
*BRAF*-K601N/E	FGFR3-K650T/M	PDGFRA-N870S
CDK-R24C/H	FLT3-I836del	PDGFRA-D1071N
*EGFR*-R108K	FLT3_2	PDGFRA-D842_H845del
*EGFR*-T263P	FLT3_3	PDGFRA-I843_D846del
*EGFR*-A289V	FLT3-D835H/D835Y	PDGFRA-S566_E571>K
*EGFR*-G598V	*HRAS*-G12V/D	PDGFRA-I843_S847>T
*EGFR*-E709K/E709H	*HRAS*-G13C/R/S	PDGFRA-D842V
*EGFR*-E709A/E709G/E709V	*HRAS*-G13V/D	*PIK3CA*-R88Q
*EGFR*-G719S/G719C	*HRAS*-Q61H	*PIK3CA*-N345K
*EGFR*-G719A	*HRAS*-Q61H/L/R/P/K	*PIK3CA*-C420R
*EGFR*-M766_A767insAI	JAK2-V617F	*PIK3CA*-P539R
*EGFR*-S768I	KIT-D52N	*PIK3CA*-E542K
*EGFR*-V769_D770insASV	KIT-Y503_F504insAY	*PIK3CA*-E545K
*EGFR*-V769_D770insCV	KIT-W557R/W557R/W557G	*PIK3CA*-Q546K
*EGFR*-D770_N771>AGG/V769_D770insASV/V769_D770insASV	KIT-V559D/V559A/V559G	*PIK3CA*-H701P
*EGFR*-D770_N771insG	KIT-V559I	*PIK3CA*-H1047R/H1047L
*EGFR*-N771_P772>SVDNR	KIT-V560D/V560G	*PIK3CA*-H1047Y
*EGFR*-P772_H773insV	KIT-K550_K558del	*PIK3CA*-G1049R
*EGFR*-H773>NPY	KIT-K558_V560del	*PIK3CA*-R38H
*EGFR*-H773_V774insNPH/H773_V774insPH/H773_V774insH	KIT-K558_E562del	*PIK3CA*-C901F
*EGFR*-V774_C775insHV	KIT-V559del	*PIK3CA*-M1043I/M1043I
*EGFR*-T790 M	KIT-V559_V560del	RET-C634R
*EGFR*-L858R	KIT-V560del	RET-C634W/Y
*EGFR*-L861Q	KIT-Y570_L576del	RET-E632_L633del
*EGFR*-L747_T750del, P ins/E746_A750del, T751A	KIT-E561K	RET-M918T
*EGFR*-E746_T751del, I ins/S752_I759del	KIT-L576P	RET-A664D

### Data analysis

Data analysis was performed using MassArray Typer Analyzer software 4.0.4.20 (Sequenom), which facilitates visualization of data patterns as well as the raw spectra. Mutations were identified in two different ways. Typer automates the identification of mutants by comparing ratios of the wild type peak to that of all suspected mutants and generates an Onco Mutation report detailing specific mutations and the ratios of wild type and mutation peaks. In addition, raw data was exported to Excel and an in-house macro was used to duplicate the analysis. The area under the peaks allows for quantification for each allele, giving a direct evaluation of the proportion of mutated and wildtype (wt) allele in the sample [18].

All mutations from both the Onco mutation report and the in-house Excel report were reviewed manually by 3 investigators (DF, PGG, KPG). Manual review of mutations was necessary to identify "real" mutant peaks from salt peaks or other background peaks. Selected reviewed mutations from the Onco Mutation Report and from the in-house macro were compared and were concordant.

## Results

### Mutations were detected in control DNAs from intact and FFPETsamples

Previously described mutations in control cell lines were detected. *BRAF*_V600E, *HRAS*_G12D, *NRAS*_Q61L, *PIK3CA*_E545K, *KRAS*_G13D, *NRAS*_Q61K, *EGFR*1_S125L, and *PIK3CA*_H1047R were detected in the appropriate cell lines (A2058, HS578T, HL60, MCF7, MDAMB231, NCI-H1299, NCI-H1395, and UACC-893, respectively). The appropriate mutation was found in MCF-7 (*PIK3CA*_E545K) from both intact DNA and DNA isolated from FFPET. DNAs from clinical samples, control cell lines, and cell lines formalin-fixed, paraffin-embedded cell lines showed the same rates of primer extension and performance on mass spectrometer.

**Figure 2:**
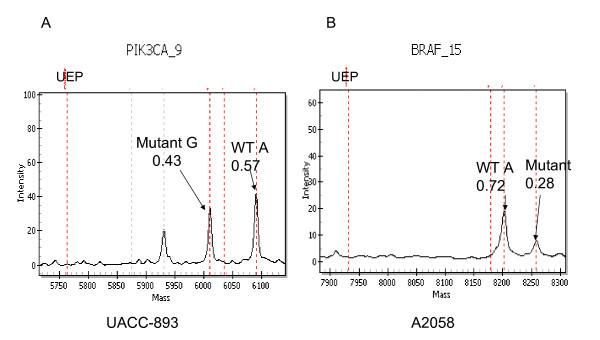
**Spectra for cell lines UACC-893 and A2058**. The expected positions for the unexteneded primer (UEP), and the extension products (Mutant and WT) from assays *PIK3CA*_9 and *BRAF*_15 in cell lines UACC-893 and A2058, respectively, are indicated with red dashed lines. The proportion of peak areas and the specific base is also shown. Assays *PIK3CA*_9 and *BRAF*_15 detected mutations in *PIK3CA *at amino acid position 1047 and in *BRAF *at amino acid position 600, respectively. Other peaks included in these spectra as result of multiplexing but not part of the designated assays are indicated as grey dashed lines.

The proportion of the mutated alleles in each cell line, as observed from the area under the mutant peak on the spectra, ranged from 0.4-0.6, as expected for a pure clonal population with a heterozygote mutation. Spectra for cell line UACC-893 had equal fractions of mutant and wt alleles (Fig. 2A). One exception to this distribution among cell lines was seen in A2058, which showed spectra consistent with 2 copies of the WT allele and one mutant *BRAF *mutant allele (Fig. 2B). The 3 alleles of *BRAF *in A2058 are consistent with the observation that there are 3 copies of chromosome 7 in this cell line (COSMIC in the SNP Array Based LOH and Copy Number Analysis data base) [21].

### The Sequenom platform was sensitive and quantitative

**Figure 3:**
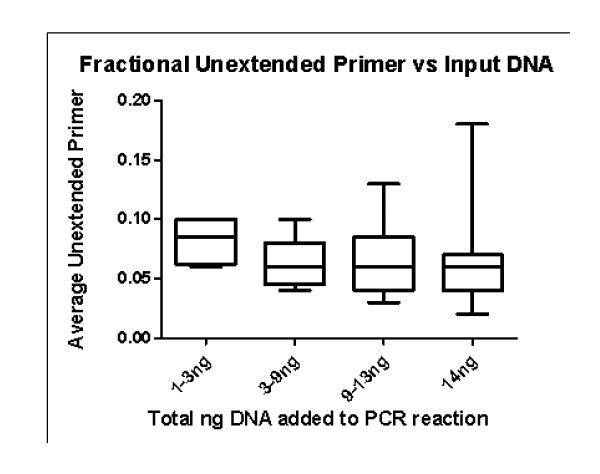
**Fractional unextended primer versus input DNA**. The range and the average for the percent of unextended primer for different amounts of input DNA into the PCR reactions are shown. The number of samples used in each category was 4 for 1-3 ng, 9 for 3-9 ng, 13 for 9-13 and 210 for 14 ng.

Pilot studies demonstrated that the assays worked with as little as 1 ng of DNA (Fig. 3). The fraction of unextended primer was .09 even when the input DNA was between 1-3 ng, When concentrations of the amount of DNA was between 3-14 ng, the fraction of unextended primer was similar, .07. Thus, the assays worked well even when only 1 ng of DNA was used.

**Figure 4:**
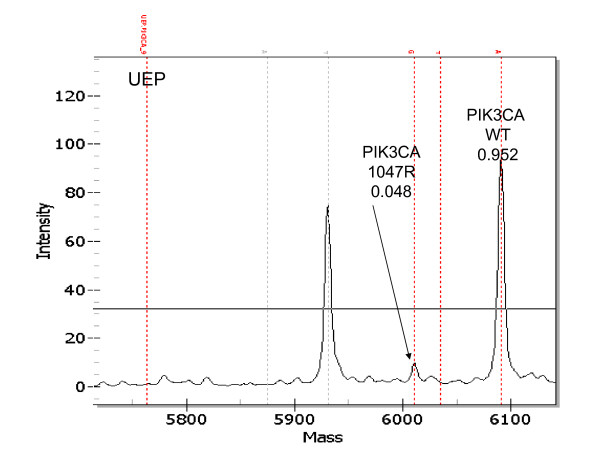
**Sensitive detection of mutations in clinical FFPE samples with the Sequenom platform**. Small mutant but definitive peak illustrating a *PIK3CA*-1047R mutation in approximately 5% of the sample DNA is shown.

**Figure 5:**
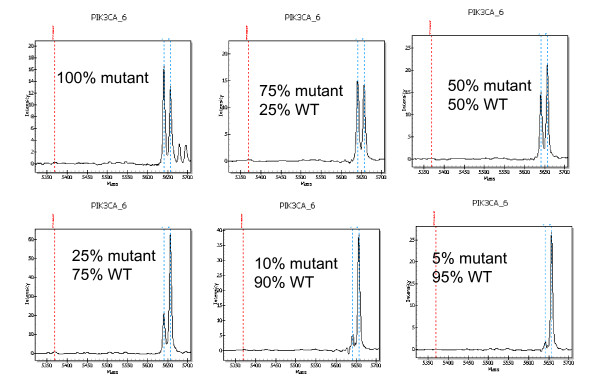
**Quantification of the sensitivity with a cell line mixing experiment**. Spectra of MCF-7 cells (mutant) alone or mixed with SKBR3 cells (WT) are shown. Percents are based on the ng amounts of DNA. This assay detects an E545K mutation in PIK3CA.

In clinical samples with some assays it was possible to detect mutations that only represented 5% of the total 2 peak areas. The spectra in Fig. 4 show a small but clear peak at the expected size for a *PIK3CA *1047R mutation in a lymph node. We also were able to demonstrate the sensitivity of the platform by performing a cell mixing experiment. Mutation analysis was done using MCF-7 cell line DNA alone or mixed with SKBR3 at various percentages. MCF-7 cells contain a PIK3CA mutation, and SKBR3 cells do not. Fig. 5 demonstrates that the mutation was detectable even when the MCF-7 cells represented only 5 to 10% of the total DNA and only 5 to 2.5% of the alleles. This sensitivity is important for mutation detection in clinical cancer samples, which usually contain some amount of normal tissue, which dilutes the number of tumor cells. This is of particular concern when profiling lymph nodes, which may contain a minority of tumor cells.

### Frequencies of C-07 mutations in *KRAS*, *NRAS*, *PIK3CA*, and *BRAF *detected with OncoCarta and the Sequenom platform were similar to previous reports

In this preliminary assessment of the feasibility of using the Sequenom platform to do large-scale mutation profiling of colon cancer samples isolated from FFPET, it was essential to determine if our data yielded frequencies typical of what has been seen previously. Table 2 shows the mutation frequencies obtained here and from the COSMIC (Catalog of Somatic Mutations in Cancer) database [21]. The COSMIC frequencies seen in Table 2 are based only on those mutations that are detectable with OncoCarta. OncoCarta assays interrogate 99%, 98%, and 78% of the known colon cancer mutations in *BRAF*, *KRAS*, and *PIK3CA*, respectively, based on a large number of colon cancer samples that have been sequenced in *BRAF *(n = 4628), *KRAS *(n = 858) and *PIK3CA *(n = 247). The OncoCarta panel found that the most frequent mutations in C-07 were *KRAS *(43.5%), *PIK3CA *(20.1%), and *BRAF *(12.1%), which are similar to what is seen in COSMIC. *NRAS *mutations, while infrequent, were detected in codons 12, 13 and 61 and represent a sizable minority of the C0-7 samples (3.8%). These data suggest that FFPET samples can be interrogated with the technology described here and yield accurate data.

**Table 2:** Frequency of colon cancer mutations

Mutation	No. Mutated Samples	Frequency in Primary Tumor*	in COSMIC^†^	Multiple Mutations^‡^
*ABL1*-F359V	1	0.40%	0/66^§^	
**ABL1 Total**	**1**	**0.40%**	NF(0/66)^§^	100%
*AKT1*-E17K	1	0.40%	0/31^§^	
**AKT1 Total**	**1**	**0.40%**	NF(0/31)^§^	100%
*BRAF*-D594V| G	1	0.40%	NF (0/3179)	
*BRAF*-V600E	28	11.70%	14.60%	
**BRAF Total**	**29**	**12.10%**	14.70%	24%
*KRAS*-G12A	2	0.80%	1.80%	
*KRAS*-G12C	10	4.20%	3.60%	
*KRAS*-G12D	40	16.70%	13.20%	
*KRAS*-G12R	3	1.30%	0.40%	
*KRAS*-G12S	3	1.30%	4.20%	
*KRAS*-G12V	20	8.40%	7.40%	
*KRAS*-G13D	23	9.60%	5.20%	
*KRAS*-A59T	1	0.40%	0.10%	
*KRAS*-Q61L	1	0.40%	0.20%	
*KRAS*-Q61R	1	0.40%	NF (0/1927)	
**KRAS Total**	**104**	**43.50%**	36.10%	34%
*MET*-R970C	2	0.80%	NF (0/77)	
*MET*-T992I	6	2.50%	NF (0/77)	
**MET Total**	**8**	**3.30%**	0%	50%
*NRAS*-G12C	1	0.40%	NF (0/46)	
*NRAS*-G12D	4	1.70%	NF (0/46)	
*NRAS*-G13R	1	0.40%	NF (0/46)	
*NRAS*-G13V	1	0.40%	NF (0/46)	
*NRAS*-Q61H	1	0.40%	NF (0/46)	
*NRAS*-Q61K	1	0.40%	NF (0/46)	
**NRAS Total**	**9**	**3.80%**	2.2%	38%
*PIK3CA*-R88Q	5	2.10%	NF (0/171)	
*PIK3CA*-C420R	2	0.80%	NF (0/171)	
*PIK3CA*-E542K	9	3.80%	4.10%	
*PIK3CA*-E545K	12	5.00%	4.10%	
*PIK3CA*-Q546K	4	1.70%	1.20%	
*PIK3CA*-H701P	1	0.40%	NF (0/171)	
*PIK3CA*-H1047L	1	0.40%	1.80%	
*PIK3CA*-H1047R	14	5.90%	5.30%	
**PIK3CA Total**	**48**	**20.10%**	16.40%	80%

*% of C-07 samples with this mutation.

^†^Data from COSMIC for colon adenocarcinoma limited to the same mutations interrogated with OncoCarta. The mutations listed are only the ones found in C-07. Some COSMIC amino acid changes are not shown here if they were not mutated in C-07.

^‡^% of samples with a mutation in the gene shown and at least one other mutation in C-07 samples.

^§^COSMIC data are from large intestine, not specific to colon.

While most of the specific amino acid mutations mirror what is seen on the COSMIC database, some unique colon cancer gene mutations were found, which include *ABL1*-F359V, *AKT1-*E17K, *MET-*R970C, and *MET*-T992I. Other amino acid changes that were not in the COSMIC database were amino acid changes R88Q, H701P, and C420R in *PIK3CA*, *BRAF*-594V/G, and *KRAS*-Q61R, and several in *NRAS*, including G12C, G12D, G13R, G13V, Q61H and Q61K (Table 2).

### MET mutations were found in C0-7 and amplified in sometumors

**Figure 6:**
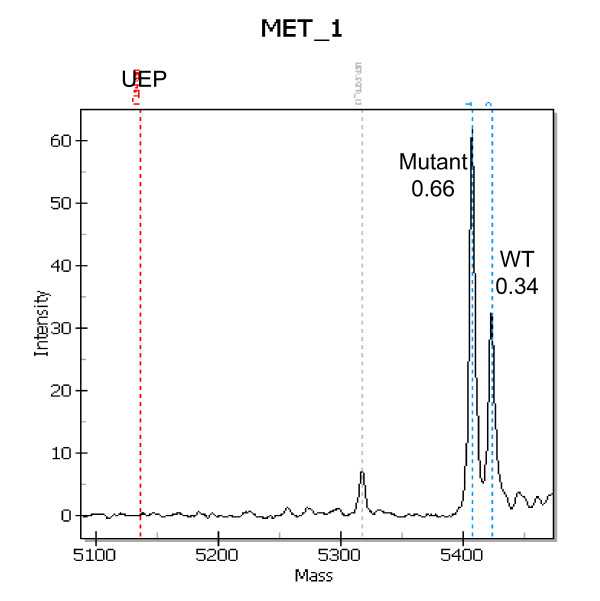
**MET mutation is amplified**. The proportion and position (blue dashed lines) of mutant and wt alleles are shown. The *MET*_1 assay detects R970C mutations in *MET*.

*MET *mutations were found in 3.3% of C-07 samples. Interestingly, these mutations were not only unexpected in their appearance within the colon cancer population but also the frequency within the samples was unexpected. In four of the eight samples with *MET *mutations, the mutant alleles were present at 58-70%, suggesting an amplification of the mutant allele or a loss of the wt gene (Fig. 6). Amplification may represent the best explanation, in that amplification of the *MET *genomic region, 7q31, has been observed in the Progenetix CGH Database in 23% of colorectal cancers [22]

### Sequenom data was reproducible

Most of the assays in the OncoCarta panel did not detect mutations or the frequency of mutations was very low (below 1%) in our colon cancer samples. OncoCarta assays interrogate mutations in these 19 genes listed in Table 1. To reduce the cost, time and the amount of DNA required for profiling, only 24 assays, which detected mutations at a frequency of 1% or greater in C-07, were selected, resorted in 6 pools and included in a new panel, termed ColoCarta (Table 3). Mutation profiles of 32 mutant samples with 41 mutations were repeated with the ColoCarta. The mutations detected by the 2 panels (OncoCarta and ColoCarta) were identical, demonstrating the reproducibility of the methodology.

**Table 3:** ColoCarta panel

Sequenom's Assay Name	Amino Acid Change
*BRAF*_15 &16	V600E/K/R/L
*BRAF*_9	*BRAF*-D594V
*HRAS*_6*	*HRAS*-Q61L
*KRAS*_1 & 2	G12V/A/D/C/S/R/F
*KRAS*_4	*KRAS*-G13D
*KRAS*_5	*KRAS*-A59T
*KRAS*_7	*KRAS*-Q61L
*KRAS*_8	Q61H/Q61H
*MET*_1	*MET*-R970C
*MET*_2	*MET*-T992I
*NRAS*_1	*NRAS*-G12V
*NRAS*_2	*NRAS*-G12C
*NRAS*_3	*NRAS*-G13V
*NRAS*_4	*NRAS*-G13C
*NRAS*_7	*NRAS*-Q61H
*NRAS*_8	*NRAS*-Q61E
*PIK3CA*_1	*PIK3CA*-R88Q
*PIK3CA*_3	*PIK3CA*-C420R
*PIK3CA*_5	*PIK3CA*-E542K
*PIK3CA*_6	*PIK3CA*-E545K
*PIK3CA*_7	*PIK3CA*-Q546K
*PIK3CA*_8	*PIK3CA*-H701P
*PIK3CA*_9	*PIK3CA*-H1047R

*HRAS_6 was included in panel but occurred in < 0.1% of samples.

### Multiple mutation frequencies suggest an order to the acquisition of different mutations

A majority of the tumors (64%) contained at least one or more mutations in the following genes: *BRAF*, *KRAS*, *NRAS*, *MET*, or *PIK3CA*, and 18% had 2 or more mutations. The most common double mutation was in *KRAS *and *PIK3CA*, followed by *PIK3CA *and *BRAF *(Table 4). Most samples with *PIK3CA *mutations (80%) also had mutations in other genes, the most frequent of which was *KRAS*; other mutated genes were *BRAF*, *MET*, *NRAS*, and a second *PIK3CA *mutation (Table 2, last column). Tumors with *MET *and *NRAS *mutations also have an unexpectedly high frequency of co-occurring mutations, which suggests that they occur as a second mutation and perhaps later in the etiology of the tumor. Many tumors contain only a *KRAS *or *BRAF *mutation, which is consistent with previous reports finding these mutations in earlier stages of colon cancer [23,24]. The multiple mutation frequencies for tumors with *KRAS *and *PIK3CA *or with *PIK3CA *and *BRAF *were slightly higher and lower, respectively, than expected based on their individual frequencies (Table 4). Conversely, the expected double mutation frequency of *BRAF *and *KRAS *would be 5.1%, based on our data, but this combination was not found, also in agreement with previous reports [24] (Table 4).

**Table 4:** Single and double mutations in C-07

	Double Mutation Frequencies						
		KRAS		PIK3CA		All other	
	Single	Actual	Expected	Actual	Expected	Actual	Expected
KRAS	43.70%	NA	NA	10.40%	8.70%	14.60%	7.21%
PIK3CA	20.10%	10.40%	8.70%	NA	NA	15.50%	8.06%
BRAF	11.80%	0	5.10%	1.80%	2.40%	2.50%	5.71%
MET	3.30%	1.67%	1.44%	0	0.66%	1.67%	2%
NRAS	3.80%	0	1.66%	0.42%	0.40%	1.30%	2.14%
All Mutations	60.20%						

### Primary tumors with KRAS and PIK3CA mutations vary with respect to the frequency of these mutant alleles

In the samples with co-occurring mutations, the ratios of *KRAS *mutation ratio (*KRAS *mutation peak area/total peak area) to the *PIK3CA *mutation ratio (*PIK3CA *mutation peak area/total peak area) was determined. Twenty-two out of 31 samples (71%) had *KRAS*/*PIK3CA *ratios above 1.25 (Table 5). *PIK3CA *mutations were more prevalent in only 2 out of 31 samples. These differences demonstrate that in a majority of primary tumors with double mutations in *KRAS *and *PIK3CA*, the *KRAS *mutations are more prevalent than the *PIK3CA*. This unequal distribution of mutant alleles within a tumor may be due to the fact that a majority of the tumor cells have only the *KRAS *mutation, and cells with a *PIK3CA *mutation are in the minority, or it could be due to copy number variations in the *KRAS *and *PIK3CA *loci.

**Table 5:** KRAS/PIK3CA ratio mutation frequencies within primary tumors

No of Samples	KRAS/PIK3CA
22	1.25-3.22
7	0.93-1.13
2	0.81-.42
**Average**	1.67
**Median**	1.6

### BRAF mutations were correlated with poorly differentiated tumors and with mucinous tumors

The frequency of mutations for *KRAS*, *PIK3CA*, and *BRAF *were tested for correlation to the degree of differentiation and to the prevalence of mucin in the tumor. *BRAF *mutations were found in 26.2% of the poorly differentiated tumors and in 8.2% of the moderate and well differentiated. These frequencies were significantly different by Chi square test (p value = 0.001). *BRAF *mutations were also associated with mucinous tumors: *BRAF *mutations occurred in 28% of grade 3 mucinous tumors (>50% mucinous tumor cells) but in only 9.4% of the non-mucinous tumors (grade 1 and 2). This was significant by the Chi square test at p value = 0.006. Similar data have been reported previously [25,26]. *KRAS *and *PIK3CA *mutations did not correlate with either the degree of differentiation or with prevalence of mucinous cells.

### Mutation profiling demonstrated a majority of primary and lymph node samples were concordant but differences were detected

Lymph node metastases were not routinely collected in C-07 but as a pilot study to determine the feasibility of using lymph nodes for mutation profiling was conducted. We isolated DNA from 39 lymph nodes containing tumor cells and their corresponding primary tumors. These primary and lymph nodes samples were profiled with the entire OncoCarta panel. The majority of lymph nodes and their corresponding primary tumors (89.7%) were concordant. A total of 26 mutations were detected in lymph nodes, including *KRAS*, *BRAF*, *PIK3CA*, and *NRAS*. Thirty-five out of 39 lymph nodes had identical mutation profiles, but in 4 cases mutations in the primary tumors were not found in the corresponding lymph nodes (*BRAF *[2], *PIK3CA *[1] and *KRAS *[1]).

### Mutation profiles demonstrate that tumor cell populations may be different in lymph nodes and in the primary tumors

Peak area evaluation of tumors that had 2 mutations and for which a metastatic lymph node was available demonstrated differences between the primary and lymph node samples. Table 6 details the frequency of mutant and wt alleles based on the peak areas for 5 such samples.

**Table 6:** Allele frequencies for primary tumors with two mutations and metastatic lymph nodes

Sample	Mutations	Mutant Allele Frequency		Mutation Ratios	
		Primary	Lymph Node	Primary M1/M2	Lymph node M1/M2
C07-0388	M1. *KRAS*-G12D	0.45	0.44	1.88	1.63
	M2. *PIK3CA*-H1047R	0.24	0.27		
C07-0717	M1. *KRAS*-G13D	0.08	0.09	1.14	0.90
	M2. *PIK3CA*-H1047R	0.07	0.1		
C07-0940	M1. *KRAS*-G12D	0.42	0.34	1.91	1.10
	M2. *PIK3CA*-E542K	0.22	0.31		
C07-2244	M1. *KRAS*-G12C	0.37	0.15	1.76	_
	M2. *PIK3CA*-H1047R	0.21	0		
C07-1837	M1. *BRAF*-V600E	0.2	0.22	1.67	4.40
	M2. *PIK3CA*-H1047R	0.12	0.05		

*KRAS *to *PIK3CA *ratios demonstrated that there were more *KRAS *mutations than *PIK3CA *mutations in 4 of 4 primary samples, and in 3 of the 4 lymph node samples. However, it is also notable that the ratio of *KRAS*/PIK3A was lower in the lymph node compared to their primary tumor in 3 out of 4 samples. In sample 0940, the *KRAS*/*PIK3CA *mutation decreased by almost 1/2 in the lymph node tumor compared to the primary. Thus, in these samples there is either a loss of *KRAS *mutations or an accumulation of *PIK3CA *mutations, suggesting that *PIK3CA *mutations may impart a selective advantage in the lymph node.

In contrast, two other samples have a less frequent occurrence of their *PIK3CA *mutation in the lymph node than in the primary tumor. In sample 2244, the *PIK3CA *mutation was undetectable in the lymph node (Table 6). In fact, if there was a selection for both mutations in the lymph node, then the *PIK3CA *mutation frequency would have been the same as that of the *KRAS *mutation (0.15). On the other hand, if the *PIK3CA*/*KRAS *ratio were the same in the primary and lymph node tumor, then the *PIK3CA *mutation frequency would have been .08, which is still detectable with this technology (Fig. 3). Thus, in sample 2244 there were fewer *PIK3CA *mutant alleles in the lymph node than in the primary tumor. In sample 1837, mutations in both *BRAF *and *PIK3CA *were detected and the *BRAF*/*PIK3CA *ratio was 1.67, but increased to 4.4 in the metastatic lymph node.

## Discussion

The Sequenom platform provides a superior technology for the screening of many hot spot mutations in cancer samples. Sanger sequencing would require amplification of at least 60 different fragments per sample, and many reactions would require optimization, thus adding considerable time and expense. Multiplexing and the use of the OncoCarta panel allowed us to skip this time consuming step. Thus, conservatively, Sanger sequencing would be 40 times more expensive, and require at least 2 times more DNA. Other sequencing technologies, which employ differential melting of mutant and wt sequences, such as HRMA, still require that the PCR product be sequenced. This would add significant cost and time to the procedure because 60% of the colon samples contained one or more mutations. In addition the Sequenom platform is more sensitive than Sanger sequencing in that it was able to detect mutations that represented only 5% of the DNA. Pyrosequencing represented a potential alternative to the Sequenom platform, but in our hands assays needed to be optimized, and the lack of multiplexing made the procedure more time consuming and demanded more DNA. The Sequenom methodology also focuses on only those nucleotides that are known to be cancer mutations and thus makes review of the sequence information considerably faster than Sanger. Next-Generation sequencing was cost prohibitive and has not been shown to work with DNAs isolated from FFPET. Thus, the Sequenom platform and the OncoCarta Panel provided the simplest, most rapid, sensitive and cost-effective method for detecting hot spot cancer mutations in degraded DNAs isolated from archival and routinely processed FFPET. The ColoCarta panel provides a more specific panel for colon cancer mutation detection and greatly reduces the amount of DNA needed for mutation profiling.

The frequencies and specific amino acid mutations detected here were similar to the COSMIC database and other publications [6]. The small variation in frequency between our data and other reports may be attributed to differences in the stage of the samples analyzed, the number of samples considered, and the sensitivity of the technology [18]. These observations, combined with the perfect match that we obtained between the expected and the detected mutations in our control cell lines, both fresh and FFPE, and the fact that mutations detected with OncoCarta and ColoCarta were identical, suggest that the technology is reliable and reproducible in DNAs isolated from FFPE samples.

In our study, the majority of tumors (60.3%) had one or more mutations in *KRAS*, *PIK3CA*, and *BRAF*. Mutations in these genes are likely to perturb many different and overlapping signaling pathways, including *PI3K/AKT, ERK/MAPK, SAPK/JNK, NFKβ*, and others. We were also able to detect other less frequent mutations that are likely to perturb the same pathways and these may cause resistance to EGFR-targeted therapies, as reported for *KRAS*, *PIK3CA *and *BRAF*. For example, *AKT1 *and *NRAS *are molecules that are downstream mediators of the EGFR signaling pathway, and mutations in these genes are likely to affect the response to drugs that target EGFR.

Mutations in ABL, *AKT1*, and *MET *were detected here but were not listed in COSMIC, probably due to the small number of samples analyzed. The *AKT1*-E17K mutation was initially identified as a SNP, rs34409589, but in a recent publication it was found to be a somatic mutation and was found in 3 of 51 colon cancers [27]. The frequency of these mutations in this small study (51 samples) was 6% and is much greater than in the C0-7 samples (0.4%). This difference in frequencies may be because the Carpten et al [27] samples were from more advanced stages than those from the C-07 trial. Moreover, they selected large tumors (>100 mg) and containing more than 60% tumor cells. No such selection was done for our study, and samples were from stages II and III exclusively. The significance of *ABL1 *and *AKT1 *mutations for patient prediction and prognosis in our study is questionable given that they each were found in only in one sample and represented only 0.4% of the cases.

To our knowledge, this is the first report of *MET *mutations in the primary colon cancer, but a different *MET *mutation (N1118Y) was found in a lung metastasis of the large intestine [28]. The *MET *mutations, R970C and T992I, were detected in 8 out of 239 C-07 colon cancers. These mutations correspond to *MET*-R988C and *MET*-T1010I, respectively, in the long form of MET which is the isoform referred to in the COSMIC database [29]. The R970C and T992I mutations are located in the juxtamembrane segment of the protein and were detected in lung carcinoma [30]. These mutations, when introduced into a lung cell line, increased focus formation, formation of colonies in soft agar, cell motility, and migration. These mutations also resulted in constitutive tyrosine phosphorylation on several cellular proteins including paxillin at key tyrosine residues and may account for the increased motility of cells with this mutation. Another critical amino acid in this location is a Ser 985, which, when phosphorylated, has been found to diminish *MET *signaling [31]. If phosphorylation at Thr residue 992 (1010) reduces signaling, then the R992I mutation would inhibit this negative feedback and may result in constitutive signaling [30].

If *MET *mutations confer an alternative activated signaling pathway, then these mutations could also confer resistance to anti-EGFR-based therapies or provide a new target for directed therapies. Therapeutic drugs have been developed to specifically target *MET*, including small molecule kinase inhibitors, anti-*MET *monoclonal antibodies, and inhibitors of HGF, the *MET *ligand. Invitro assays have demonstrated that a number of *MET *targeted therapies were able to prevent *MET *signaling, decrease cell viability, and limit cell motility and migration in vitro [32]. The small molecule ARQ 197, a kinase inhibitor, has entered phase II clinical trials so may represent a possible therapeutic strategy for some colon tumors.

To our knowledge, this is also one of the most exhaustive analyses of mutation profiling of metastatic lymph nodes and their corresponding primary colon tumors. Our analysis showed that a majority of samples were concordant (89.7%) but in a few samples mutations were detected only in the primary tumor and not in the metastatic lymph node. Also in samples with 2 co-occurring mutations, the ratio of the double mutations varied in primary and lymph node tumors. Discordance in the genetic profile between primary tumors and the metastatic lymph nodes has been observed [33]. Such data may indicate that tumor cell migration selects different cell populations from the one in the primary tumor. However, it is also possible that these mutational differences between the lymph node and the primary tumor are a result of tumor heterogeneity.

Another interesting observation in our study was that *BRAF *mutations were significantly correlated with poorly differentiated tumors and the prevalence of mucin; similar observations have been reported [25,26]. These characteristics are both associated with a worse prognosis and are consistent with other reports associating *BRAF *mutations with a bad prognosis [3]. However, in our study we found that there were 2 metastatic lymph nodes that did not maintain the *BRAF *mutation present in the corresponding primary tumor, suggesting that *BRAF *mutations are not essential for metastatic spread to the lymph node in all tumors. Clearly, additional studies would be required to understand these apparent inconsistencies; additional lymph node samples are not currently available but could be the subject of further studies when samples become available [3].

## Conclusions

The Sequenom platform provided a superior technology for the screening of 238 common hot spot cancer mutations in 19 genes. The frequent occurrence of *KRAS*, *PIK3CA*, and *BRAF *was confirmed, and mutations not detected before in colon cancer were found in *MET *and *ABL1*. Twenty-five assays from the OncoCarta were replexed to form a new panel, termed ColoCarta, which will be used to screen an additional 800 tumors from NSABP clinical trial C0-7 with the purpose of identifying prognostic or predictive markers for stage II and III colon cancer.

**Note Added in Proof: **Although MET-R988C and MET-T1010I mutations were listed in COSMIC as somatic cancer mutations, these nucleotide changes correspond to SNPs rs34589476 and rs56391007 in the NCBI SNP data base, respectively. The frequency for these SNPs is unknown so whether these nucleotide changes are associated with cancer is unknown.

## Competing interests

The authors declare that they have no competing interests.

## Authors' contributions

DF and PGG designed and carried out the experiments and participated in the drafting of the manuscript. YT graded tumors with regard to the degree of differentiation, mucin content, and defined tumor regions, S-IK carried out experiments. H-JC defined the tumor regions; SP participated in the coordination of the study; KLP-G designed and coordinated the study and drafted the manuscript. All authors have given final approval of the version to be published.

## Pre-publication history

The pre-publication history for this paper can be accessed here:

http://www.biomedcentral.com/1471-2407/10/101/prepub
